# Selection of Candidate Housekeeping Genes for Normalization in Human Postmortem Brain Samples

**DOI:** 10.3390/ijms12095461

**Published:** 2011-08-26

**Authors:** Ilaria Penna, Serena Vella, Arianna Gigoni, Claudio Russo, Ranieri Cancedda, Aldo Pagano

**Affiliations:** 1 Oncology, Biology, and Genetics Department (DOBiG), University of Genoa, Largo R. Benzi, 10, 16132 Genoa, Italy; E-Mails: iaiap84@alice.it (I.P.); serena_v@msn.com (S.V.); arianna.gigoni@hotmail.it (A.G.); ranieri.cancedda@unige.it (R.C.); 2 Department of Health Sciences, University of Molise, Via De Sanctis, 86100 Campobasso, Italy; E-Mail: claudio.russo@unimol.it; 3 National Institute for Cancer Research (IST) Genoa, Largo R. Benzi, 10, 16132 Genoa, Italy

**Keywords:** Alzheimer’s disease, quantitative real-time RT-PCR, reference genes, geNorm, NormFinder

## Abstract

The most frequently used technique to study the expression profile of genes involved in common neurological disorders is quantitative real-time RT-PCR, which allows the indirect detection of very low amounts of selected mRNAs in tissue samples. Expression analysis by RT-qPCR requires an appropriate normalization to the expression level of genes characterized by a stable, constitutive transcription. However, the identification of a gene transcribed at a very stable level is difficult if not impossible, since significant fluctuations of the level of mRNA synthesis often accompanies changes of cell behavior. The aim of this study is to identify the most stable genes in postmortem human brain samples of patients affected by Alzheimer’s disease (AD) suitable as reference genes. The experiments analyzed 12 commonly used reference genes in brain samples from eight individuals with AD and seven controls. After a careful analysis of the results calculated by geNorm and NormFinder algorithms, we found that CYC1 and EIF4A2 are the best reference genes. We remark on the importance of the determination of the best reference genes for each sample to be analyzed and suggest a practical combination of reference genes to be used in the analysis of human postmortem samples.

## 1. Introduction

Alzheimer’s disease (AD) is a progressive neurodegenerative pathology affecting hippocampus, temporal and/or frontal cerebral cortex lobes. It causes a selective loss of cortical neurons leading to the intracellular deposition of Tau (microtubule-associated protein tau NM_001123066.3) protein neurofibrillary tangles and to the extracellular deposition of β-amyloid peptide (Aβ) in form of senile plaques [1]. One of the main concerns of AD research practices is the lack of sources of experimental samples. Indeed, the available brain samples derive solely from autopsies and, as a consequence, carry several limitations. Thus, the low quality of RNA from autopsy samples has been always an important issue, since the vast majority of experiments involve the analysis of genes expression [2]. In this context the most frequently used approach to study the expression profile of genes involved in AD is quantitative real-time RT-PCR (RT-qPCR), which allows the indirect detection of very low amounts of selected messenger RNAs in tissue samples. Unfortunately, the identification of a gene transcribed at a stable level is very difficult if not impossible since significant fluctuations of the level of synthesis often accompany changes of cell behavior [3–5]. Thus, an ideal reference gene for data normalization would be consistently expressed in all samples in any experimental condition, regardless of the tissue type and possible disease state whereas its quantitative expression should be comparable to that of the target gene [4,6]. In this context it is reasonable to conclude that there is not a universal reference gene suitable for different experimental conditions and that individual cases should contemplate a careful evaluation and validation of several reference genes prior to choosing the appropriate one [6,7]. Indeed, alterations of the reference gene expression between target samples and their controls may lead to a significant alteration of the results [8].

The vast majority of RT-qPCR expression profiles found in the literature (including our recent experiments) uses GAPDH (glyceraldehyde-3-phosphate dehydrogenase, NM_002046.3) as a reference gene for normalization. In the specific case of neurodegeneration, a series of contributions aimed to determine the suitability of this gene demonstrated an abnormal aggregation of GAPDH protein product in the nucleus of cells from AD patients together with an increased activity of this enzyme in the diseased tissues, keeping in line with a direct and/or indirect correlation of GAPDH with the neurodegenerative process [9,10]. On the contrary, although neither alterations of the enzymatic activity nor aberrant capacity to aggregate directly affect RT-qPCR determinations, the mRNA level has been found to be slightly decreased in AD tissues with respect to their non-diseased counterparts. Therefore, altogether a decreased synthesis of GAPDH mRNA, an increased aggregation of GAPDH protein and an augmented activity of GAPDH enzymatic activity reported in neurodegenerated tissues suggest a possible involvement of this enzyme in neurodegenerative pathologies and suggest the use of a different reference gene in these samples.

In this work we report the comparative analysis of 12 different genes as putative reference genes for postmortem AD samples and controls in RT-qPCR practices and provide evidence for the usefulness of CYC1 and EIF4A2 in these experiments, excluding specific references with bad performances.

## 2. Results

In order to compare expression levels of target genes in different tissues at the same time, it is crucial to normalize all samples by the same set of reference genes. For the evaluation of potential references, we used the geNorm Housekeeping Gene Selection Kit (PrimerDesign-Eppendorf, Hamburg, Germany) to evaluate 12 commonly used reference genes in different human brain samples from 8 postmortem cerebral cortices of individuals with AD and 7 controls (Table 1).

As shown in Table 2, the reference genes tested were RN18S1 (18S ribosomal RNA subunit), ACTB (beta-actin), TOP1 (DNA topoisomerase type I), ATP5B (ATP synthase, H+ transporting, mitochondrial F1 complex, beta polypeptide), B2M (beta-2 microglobulin), CYC1 (Cytochrome c1), EIF4A2 (eukaryotic translation initiation factor 4a2), GAPDH (glyceraldehyde-3-phosphate dehydrogenase), RPL13A (ribosomal protein L13a), SDHA (succinate dehydrogenase complex, subunit A), UBC (ubiquitin C) and YHWAZ (tyrosine 3-monooxygenase/tryptophan 5-monooxygenase activation protein, zeta polypeptide).

The total RNA obtained from 15 different human brain samples were analyzed for their purity and the concentration by measuring the absorbance ratio at 260/280 nm. Total RNA samples were shown to be free of protein contaminants with values between 1.6 and 2 (data not shown).

We also tested RNA integrity by electrophoresis on 2% agarose gel as indicated in the MIQE guidelines [11] (data not shown).

**Figure 1:**
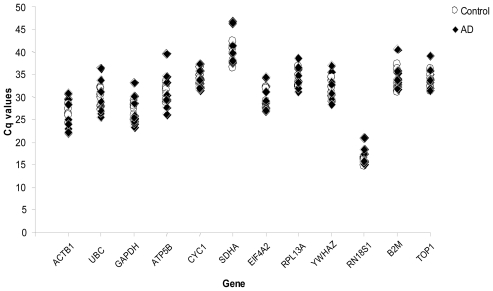
Expression levels of candidate reference genes. Values are given as quantitative real-time RT-PCR cycle threshold (Cq) values for 12 candidate reference genes in 15 human brain samples (○: Control; ◆: AD).

Equal amounts of RNA extracted from samples were reverse transcribed and used as a template for RT-qPCR according to the manufacturer’s protocol (PrimerDesign-Eppendorf). To evaluate the expression stability of the selected reference genes, the Cq values of the sample collection were averaged to obtain raw Cq values. As shown in Figure 1, the 12 HKGs demonstrated a wide range of expression level, from the lowest Cq of 17.1 for the RN18S1 (18S ribosomal RNA subunit) to the highest Cq of 46.75 for the SDHA (succinate dehydrogenase complex, subunit A). This is an ideal condition because the levels of expression of normalizers span a broad range of expression levels and should be similar to genes of interest.

The Cq values were then transformed into relative quantification data using the 2(^−deltaCq^) method and then analyzed by geNorm [12] and NormFinder [13].

**Figure 2:**
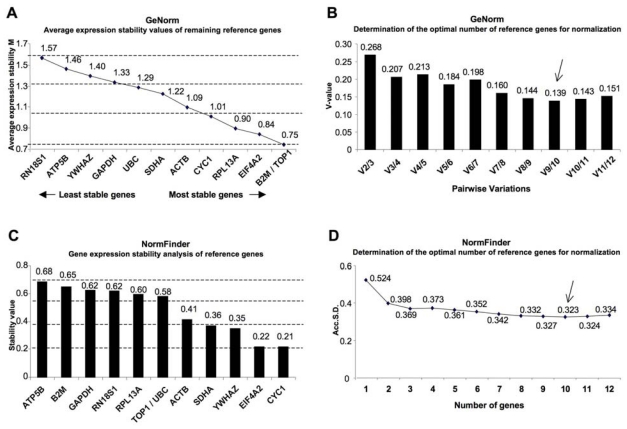
Expression stability of 12 reference genes in human brain samples. (**A**) Average expression stability values (M) of 12 candidate genes from the least stable to the most stable, calculated by geNorm; (**B**) Determination of optimal number of reference genes required for accurate normalization based on pairwise variation (V_n_/V_n+1_) between candidate genes; (**C**) Stability of different reference gene candidates according to NormFinder; (**D**) Determination of the optimal number of control genes for normalization data on the calculation of the Accumulated Standard Deviation (Acc.S.D.) using NormFinder.

GeNorm is a statistical algorithm designed to analyze the expression stability of a list of selected reference genes in all samples, and ranking them according to gene stability measurements (M). In this approach, the M value is defined as the mean pairwise variation of a gene with all the other reference genes in a given set of samples so that a low M value is indicative of more stable expression [12]. In our samples we found that the M values for the genes (from the least stable to the most stable) were the following: RN18S1, ATP5B, YWHAZ, GAPDH, UBC, SDHA, ACTB, CYC1, RPL13A, EIF4A2, TOP1 and B2M (Figure 2A and Table 2). Previous studies defined M = 1.5 as an acceptable cut-off for selection of RT-qPCR reference genes [14,15]. All the genes analyzed here reached high expression stability, with all the M values below the default limit of 1.5, with the exception of RN18S1 (M = 1.57). In this condition, with the final aim to select the best, intermediate and the worst reference genes based on their expression stability, we divided the M value range arbitrarily in three groups. As shown in Figure 2A, the most stable genes (0.75 < M value < 1.02) were CYC1, RPL13A, EIF4A2, B2M and TOP1. The intermediate stable genes (1.02 < M value < 1.29) were UBC, SDHA and ACTB, whereas the group of genes characterized by a fluctuating expression (1.29 < M value < 1.57) were RNA18S1, ATP5B, YWHAZ and GAPDH.

Next, the geNorm program was used to calculate the pairwise variation (V) among the reference genes providing an estimate of the optimum number of reference candidates to be used for data normalization (Figure 2B). The pairwise variation (V_n/n+1_) showed the effect of adding further reference genes on the normalization factor (that is calculated as the geometric mean of the expression values of the selected reference genes). When analyzing reference genes for our samples, we observed that the stepwise inclusion of individual reference genes showed an initial decrease until a minimal value (V_9/10_). The next addition of other reference genes determined an increase of V_n/n+1_, suggesting that there was a decrease of expression stability, due to the inclusion of a relatively unstable eleventh gene. This particular curve suggests that inclusion of either too few or too many reference genes could lead to erroneous normalization. Therefore, for the optimal normalization of data in this experimental setting, it would be theoretically appropriate to use 10 reference genes (BM2, TOP1, EIF4A2, RPL13A, CYC1, ACTB, SDHA, UBC, GAPDH and YWHAZ) (Figure 2B).

In order to test and/or validate our findings, the above data were analyzed by NormFinder [13]. This algorithm is based on a separate analysis of the two groups of samples (in this study, AD cases and controls) and combined the intragroup and intergroup expression variations of the selected reference genes into a stability value that ranked reference genes according to their expression stability. The candidates with the lowest intragroup and intergroup variations give the lowest stability value and are therefore ranked higher. The calculated stability values of the 12 candidate genes are reported in Table 2 and Figure 2C. Based on NormFinder, the most stable reference gene for postmortem brain samples is CYC1, followed by EIF4A2, YWAHZ, SDHA, ACTB, UBC, TOP1, RPL13A, RN18S1, GAPDH, B2M and ATP5B. In order to match the results obtained with NormFinder to those obtained with geNorm, we first divided the stability value ranges in three intervals corresponding to the most, intermediate and the least stable genes. The most stable genes (0.21 < stability value < 0.37) were SDHA, YWHAZ, EIF4A2 and CYC1. The intermediate gene (0.37 < stability value < 0.53) was ACTB, whereas the least stable genes (0.53 < stability value < 0.69) were ATP5B, B2M, GAPDH, RNA18S1, RPL13A, TOP1 and UBC.

Next, taking advantage of NormFinder, we also calculated the Accumulated Standard Deviation (Acc.S.D.) as an indicator of the optimal number of reference genes to be used in the normalization process. Results showed that the lowest value of the Acc.S.D. (indicating the optimal number of reference genes) is reached when 10 reference genes are used (0.3233) (Figure 2D).

In conclusion, the above results demonstrate that: (1) the theoretically optimal normalization is reached by using 10 different reference genes based on results from both algorithms; (2) since this number of determinations might be time consuming and expensive, the use of the combination of the two genes that fall in the best rank of both the algorithms (CYC1 and EIF4A2) are recommended as the best compromise solution for RT-qPCR determinations in human postmortem cerebral cortex samples.

## 3. Discussion

Quantitative real-time RT-PCR is a powerful and widely used technique for quantitative analysis of expression levels due to its high sensitivity, specificity, accuracy and reproducibility and to its suitability for the analysis of very small samples.

This technique requires an appropriate normalization of the data in order to minimize possible differences between test samples due to technical variations [12]. The most commonly used approach for transcript normalization takes advantage of internal reference genes characterized by a stable expression level [16]. To this aim the vast majority of studies exploits GAPDH, β-actin, or 18S rRNA, although recent experiments demonstrate that their expression can vary under different experimental conditions [4,5]. In particular, RN18S1 has been excluded in previous studies, due to its high levels of expression [6,17], because such high abundance relative to a target can make RT-qPCR data analysis difficult [12]. Therefore, these findings suggest searching for the best reference gene for each specific tissue and/or sample being studied [12].

In this work we tested several candidate reference genes, analyzing the results with two different algorithms in order to determine the most stably expressed and the optimum number of reference candidates suitable for RT-qPCR data normalization in postmortem brain tissue samples.

geNorm [12] and NormFinder [13] algorithms are now widely used and they are based on diverging mathematical approaches. geNorm analyzes the expression stability of the tested genes in all samples, and ranks them accordingly to gene stability measure (M). In contrast to geNorm, NormFinder evaluates the expression stability of each single reference gene independently from each other, and takes into account intra- and inter-group variations for normalization. This is very important in view of limited knowledge about the co-regulation of candidates in a given experimental setting.

Although, due to their distinct statistical algorithms, the two programs used gave slightly different results, both suggest the (theoretically) optimal number of ten reference genes for an accurate normalization in postmortem brain samples. Since the ordinary use of ten reference genes for this experimental setting leads to a waste of resources, we arbitrarily established cut-off scores identifying EIF4A2 and CYC1 as the best combination of stably expressed genes that fits with both the algorithms calculations.

Altogether, the above results emphasize the importance of assessing and evaluating the stability of genes for each experimental condition prior to their use as reference genes, and suggest the use of two reference genes (EIF4A2 and CYC1) for an appropriate normalization of the RT-qPCR data in human brain tissues.

## 4. Material and Methods

### 4.1. Human Brain Samples and cDNA Preparation

Frontal and temporal cortices from 8 AD (clinical history of disease; pathological diagnosis according to the Consortium to Establish a Registry for Alzheimer’s Disease (CERAD) criteria) and 7 control cases (AD excluded by clinical history and by immunohistochemical analysis) were derived from P. Gambetti and Brain Bank (Case Western Reserve University, Cleveland, OH, USA) and C. Hulette and the Joseph and Kathleen Bryan Alzheimer’s Disease Research Center (Duke University Medical Center, Durham, NC, USA). Patient characteristics are summarized in Table 1.

Small pieces (50–100 mg) of gray matter from frontal and temporal cortices were excised under sterile conditions.

Total RNAs from samples were extracted using TRIzol reagent (Invitrogen, Carlsbad, CA, USA) according to the manufacturer’s protocol and treated with DNase (Roche, Mannheim, Germany) in order to remove any trace of genomic DNA. After extraction, RNA concentration was estimated spectrophotometrically (BioPhotometer Plus-Eppendorf, Germany) at 260 nm (A_260_), and purity of the samples was assessed by the A_260_/A_280_ ratio (A_260_/A_280_ ranging within 1.6–2, as indicated by Sambrook and Russel). RNA integrity was assessed by electrophoresis on 2% agarose gel as indicated in the MIQE guidelines [11].

1 μg of RNA was then subjected to reverse transcription by Transcriptor High Fidelity cDNA Synthesis Kit (Roche, Germany). Total RNA was denatured at 65 °C for 10 min. Then it was placed on ice and supplemented with a mix containing the reverse transcriptase enzyme. Reactions were carried out at 29 °C for 10 min, at 48 °C for 1 h and at 85 °C for 5 min. cDNA was diluted ten-fold in RNase/DNase-free water and stored at −20 °C.

### 4.2. Quantitative Real-Time RT-PCR

Experiments were performed using the geNorm Housekeeping Gene Selection Kit (PrimerDesign-Eppendorf, Hamburg, Germany).

The total RNA from samples was measured by quantitative real-time RT-PCR in duplicate wells using PrimerDesign 2X *Precision^TM^* MasterMix (Eppendorf) following the manufacturer’s instructions in an ABI PRISM 7500 Fast Sequence Detection System (Applied Biosystems, Carlsbad, California). Each reaction was performed in a final volume of 20 μL containing 10 μL PrimerDesign 2X *Precision^TM^* MasterMix (Eppendorf), 1 μL Primer/probe Mix (300 nM final), 5 μL diluted cDNA and 4 μL PCR-Grade water. No template controls contained only RNase/DNase-free water. Amplifications were performed starting with a 10 min at 95 °C for the enzyme activation, followed by 50 cycles of template denaturation step at 95 °C for 15 s, data collection at 50 °C for 30 s and extension at 72 °C for 15 s. No-template control tubes (NTC), containing water instead of template mRNA, were also run under the same conditions for each gene. No product was synthesized in the NTC, so this indicated that the RT-qPCR reagents were not contaminated with the amplicon.

### 4.3. Gene Expression Stability Analysis

Data were analyzed transforming raw Cq values into relative quantification data using the deltaCq method. To achieve this, we subtracted the highest Cq value from all other Cq values for each gene measured. Hence each Cq value has been transformed into a “delta Cq” value, with the highest delta Cq value as 0. All other values are less than 0. Then for each data point, we applied the equation 2^(−deltaCq)^. Hence all data is expressed relative to the expression of the least expressed gene.

To determine the most stable reference genes, these data obtained have been converted to appropriate input files, according to the requirements of the programs, and processed by two different algorithms: geNorm v3.4 [12] and NormFinder v0.953 [13].

## Figures and Tables

**Table 1:** Description of the Control and Alzheimer’s disease (AD) subjects (sex, diagnosis, age, postmortem delay, death status, precise location of the specimen). Abbreviations: TC = temporal cortex; FC = frontal cortex; ND = not determined.

Case Number	Sex	Diagnosis	Age (Y.O.)	Postmortem Delay (Hours)	Death Status	Area
**Control**						
90–234	M	Prostatic carcinoma	77	5	Cognitively Normal	FC
CTR 3	ND	Heart Infarct	65	10	Cognitively Normal	FC
A93–300	M	Control	88	4	Cognitively Normal	TC
CTR7	ND	Lung disease	69	7	Cognitively Normal	ND
0916	ND	Control	70	ND	Cognitively Normal	ND
CTR 8	ND	Control	ND	ND	Cognitively Normal	ND
A94–207	M	Multiple infarct basal ganglia	86	4	Cognitively Normal	ND
**AD cases**						
801	ND	AD	78	ND	Dementia	ND
AD02–011	ND	AD	64	14	Dementia	ND
AD 94–382	ND	AD	81	2.05	Dementia	TC
AD 007	ND	AD	69	ND	Dementia	ND
AD171	ND	AD	87	15	Dementia	ND
969	F	AD	82	1.05	Dementia	FC
977	M	AD	80	6.03	Dementia	FC
A92–404	M	AD	69	6.05	Dementia	TC

**Table 2:** Candidate reference genes for normalization in different brain samples (AD cases and controls) ranked according to their expression stability by geNorm and NormFinder.

			geNorm		NormFinder	

Gene name	Gene symbol	Genebank accession	Ranking order	Average M value	Ranking order	Stability value
Topoisomerase (DNA) I	TOP1	NM_003286.2	1	0.75	6	0.577
Beta-2-microglobulin	B2M	NM_004048.2	1	0.75	11	0.651
Eukaryotic translation initiation factor 4A2	EIF4A2	NM_001967.3	3	0.84	2	0.218
Ribosomal protein L13a	RPL13A	NM_012423.2	4	0.9	8	0.596
Cytochrome c-1	CYC1	NM_001916.3	5	1.01	1	0.217
Actin, beta	ACTB	NM_001101.3	6	1.09	5	0.413
Succinate dehydrogenase complex, subunit A, flavoprotein (Fp)	SDHA	NM_004168.2	7	1.22	4	0.365
Ubiquitin C	UBC	NM_021009.5	8	1.29	6	0.577
Glyceraldehyde-3-phosphate dehydrogenase	GAPDH	NM_002046.3	9	1.33	10	0.623
Tyrosine 3-monooxygenase/tryptophan 5-monooxygenase activation protein, zeta polypeptide	YWHAZ	NM_001135699.1	10	1.4	3	0.347
ATP synthase, H+ transporting, mitochondrial F1 complex, beta polypeptide	ATP5B	NM_001686.3	11	1.46	12	0.683
RNA, 18S ribosomal 1	RN18S1	NR_003286.2	12	1.57	9	0.621
